# The Price Is Right: Assessing Surgical Cost Awareness of Residents and Staff Across Surgical Specialties at a Single Training Hospital

**DOI:** 10.1093/milmed/usaf595

**Published:** 2025-12-13

**Authors:** Benjamin H Baker, Matthew P Christian, Ryan C Craig, Josiah K Low, Matthew S Christman

**Affiliations:** Department of Urology, Naval Medical Center San Diego, San Diego, CA 92134, United States; Department of Urology, Naval Medical Center San Diego, San Diego, CA 92134, United States; Department of Urology, Naval Medical Center San Diego, San Diego, CA 92134, United States; Department of Urology, Naval Medical Center San Diego, San Diego, CA 92134, United States; Department of Urology, Naval Medical Center San Diego, San Diego, CA 92134, United States

## Abstract

**Introduction:**

Awareness of the cost of providing healthcare is increasingly emphasized within graduate medical education (GME). Although studies have evaluated cost awareness within single departments, few have assessed awareness across multiple specialties or training levels. We sought to determine whether surgical cost awareness varies by specialty or with career experience.

**Materials and Methods:**

Between 2022 and 2023, seven surgical departments at Naval Medical Center San Diego were surveyed. Interns, residents, and staff physicians estimated costs of common and specialty-specific disposable or consumable surgical items. Respondents were surveyed on their self-perceived cost awareness and interest in cost literacy. Accuracy was scored based on error within 50% of actual cost. One-sample Wilcoxon signed rank tests were used to compare distributions of estimates against known true costs. Multivariable logistic regression was used to identify predictors of accuracy. This project was deemed exempt by the institutional review board (NMCSD.2021.0028).

**Results:**

There were 123 respondents constituting 2,460 cost estimates (overall response rate 57%). Although most self-assessed cost knowledge was low, interest in additional education was high. Overall accuracy was 22.9%, with a median error of 156.4% (IQR 53.9-891.7%). Estimates were significantly different than true cost for all common items and many specialty-specific items. No differences in accuracy were found by age, sex, position, or experience length. Orthopedic surgery respondents were less accurate than reference general surgery respondents (OR 0.59, 95% CI 0.4-0.86, *P *= .0067).

**Conclusions:**

Cost awareness among surgical staff and trainees was low and did not improve with experience. Broad representation of all surgical specialties is a particular strength of this study, while limitations include inherent response bias and a single-payer structure which may restrict generalizability to civilian healthcare settings. Formal educational interventions should be considered to improve cost literacy as a component of systems-based practice competencies.

## INTRODUCTION

Cost-effective healthcare is a growing imperative in medical education, particularly as national healthcare expenditures continue to outpace those of other high-income countries.^1^ Efforts to balance quality and cost have led to broader incorporation of cost-awareness training within graduate medical education (GME) curricula. Despite these efforts, many clinicians remain unaware of the costs associated with routine medical care.

Over one-third of U.S. healthcare spending goes toward administrative expenses.^2^ Although structural inefficiencies drive much of this cost, physicians—even those still in training—can influence spending through awareness of resource utilization. A Yale-New Haven study found that while 95% of senior residents believed clinicians should contain costs, only 4% knew the cost of the tests they ordered.^3^ Similar studies at Children’s Hospital of Philadelphia and in orthopedic and otolaryngologic training programs have demonstrated similarly poor cost literacy among residents and attendings alike.^4–6^ Likewise, a recent cross-specialty survey reported that cost awareness of common disposable surgical supplies was severely impaired among both attendings and trainees.^7^

The Accreditation Council for Graduate Medical Education (ACGME) identifies systems-based practice as one of six core competencies for all residents.^8^ This competency includes the ability to incorporate cost awareness and risk-benefit analysis into care. Although national initiatives such as the Choosing Wisely Canada campaign seek to emphasize resource stewardship and cost-conscious care as essential parts of medical education, characterization of the true level of cost awareness amongst surgical trainees and the effects of such initiatives has largely been limited to single-department surveys with relatively small sample sizes.^9^

Given the repeatedly poor cost-awareness demonstrated in single-specialty surveys, we sought to comprehensively assess cost-awareness across all surgical departments at our institution to compare cost knowledge across specialties and by level of training. We hypothesized that otherwise undetected differences in surgical cost awareness might exist between specialties and that increased years in training or clinical practice would correlate with more accurate cost estimates.

## METHODS

This cross-sectional survey study polled resident and staff physicians across seven surgical departments at Naval Medical Center San Diego (NMCSD) between 2022 and 2023. Naval Medical Center San Diego is a large military teaching hospital with 14 ACGME-accredited residency programs and over 200 residents. We included interns, residents, and attending physicians in our target population.

Each department was surveyed during its scheduled weekly academic conference. Surveys were conducted in person and participation was voluntary. The response rate was 100% among conference attendees, with department attendance at the time of survey ranging from 50% to 90%. Department size estimates were obtained from respective clinic managers to determine estimated absolute response rates. This project was deemed exempt by the institutional review board (NMCSD.2021.0028).

### Survey Instrument

The survey was comprised of two parts. The first part included three Likert-scale questions assessing self-perceived cost knowledge (**Supplementary Materials, Page 2**). The second part asked respondents to estimate the costs of 20 disposable surgical items. The first ten were standard items asked of all participants. The remaining ten were department-specific and selected by that department’s program director to reflect commonly used items in that specialty. The ranges of cost and complexity among these department specific items were not uniform between departments as we elected to prioritize the most specialty-relevant items based on the expert opinion of each respective department head. Although no formal validation was performed, this survey was modeled after a similar instrument used in previously published literature.^7^

Estimates were compared to true cost as provided by the hospital supply department. All respondents completed both the first and second parts of the survey instrument. The primary outcome was accuracy, with an estimate within 50% of true cost considered accurate, consistent with thresholds used in prior studies.^3–7^

### Statistical Analysis

Baseline characteristics of age, sex, specialty, and position were recorded. We also designated a variable of experience length to capture total post-graduate, specialty-specific experience. For residents and interns, this was equal to postgraduate training year. For attending, this was calculated by adding number of years as an attending to length of their specialty’s residency program at NMCSD.

Estimation error was calculated for each item cost estimate as percent difference from actual cost according to the below formula:


% difference = 100 * (estimated cost—true cost)/true cost

Estimates were scored as accurate if percent difference was below 50% and inaccurate otherwise. Accuracy was calculated for each respondent and expressed as the percentage of their estimates within 50% of the true item cost. Cost estimation errors were expressed as the percent difference from actual cost.

One-sample Wilcoxon signed rank tests compared distribution of response estimates versus true costs. Multivariable logistic regression was performed to identify predictors of accuracy, with independent variables including age, sex, specialty, position, and experience length. Odds ratios (OR) and 95% CI were reported. Significance was defined as *P* < .05. Statistical analysis was performed using R (R Core Team, Vienna, Austria).

## RESULTS

A total of 123 individuals participated between all seven surgical departments, constituting 2460 cost estimates. Overall response rate was 57%, with department-specific rates ranging from 40% to 91%. Median age was 36 years (IQR 32-42 years). Most respondents were male. There was relatively even representation between residents and staff (**Table 1**).

**Table 1.** Baseline Characteristics by Department

		ENT	General surgery	OBGYN	OMFS	Ophthalmology	Orthopedic surgery	Urology	Total
Responses (% response rate)		14 (88%)	31 (47%)	14 (47%)	12 (67%)	15 (71%)	17 (40%)	20 (91%)	123 (57%)
Median age, years (IQR)		35 (32-43)	35 (32-41.5)	35.5 (30.25-47)	37 (32-41.75)	38 (35.5-42.5)	35 (31-41)	35 (31-42.25)	36 (32-42)
Sex ratio (M:F)		9:5	21:10	4:10	11:1	10:5	15:1	15:5	87:43
Position	Resident	7 (50%)	19 (61%)	7 (50%)	5 (42%)	6 (40%)	10 (59%)	11 (55%)	65 (53%)
	Staff	7 (50%)	12 (39%)	7 (50%)	7 (58%)	9 (60%)	7 (41%)	9 (45%)	58 (47%)

Values are *n* (%) unless otherwise indicated. ENT - ear, nose and throat; OBGYN - obstetrics and gynecology; OMFS - oral maxillofacial surgery; IQR - interquartile range; M - male; F - female

**Figure 1.**
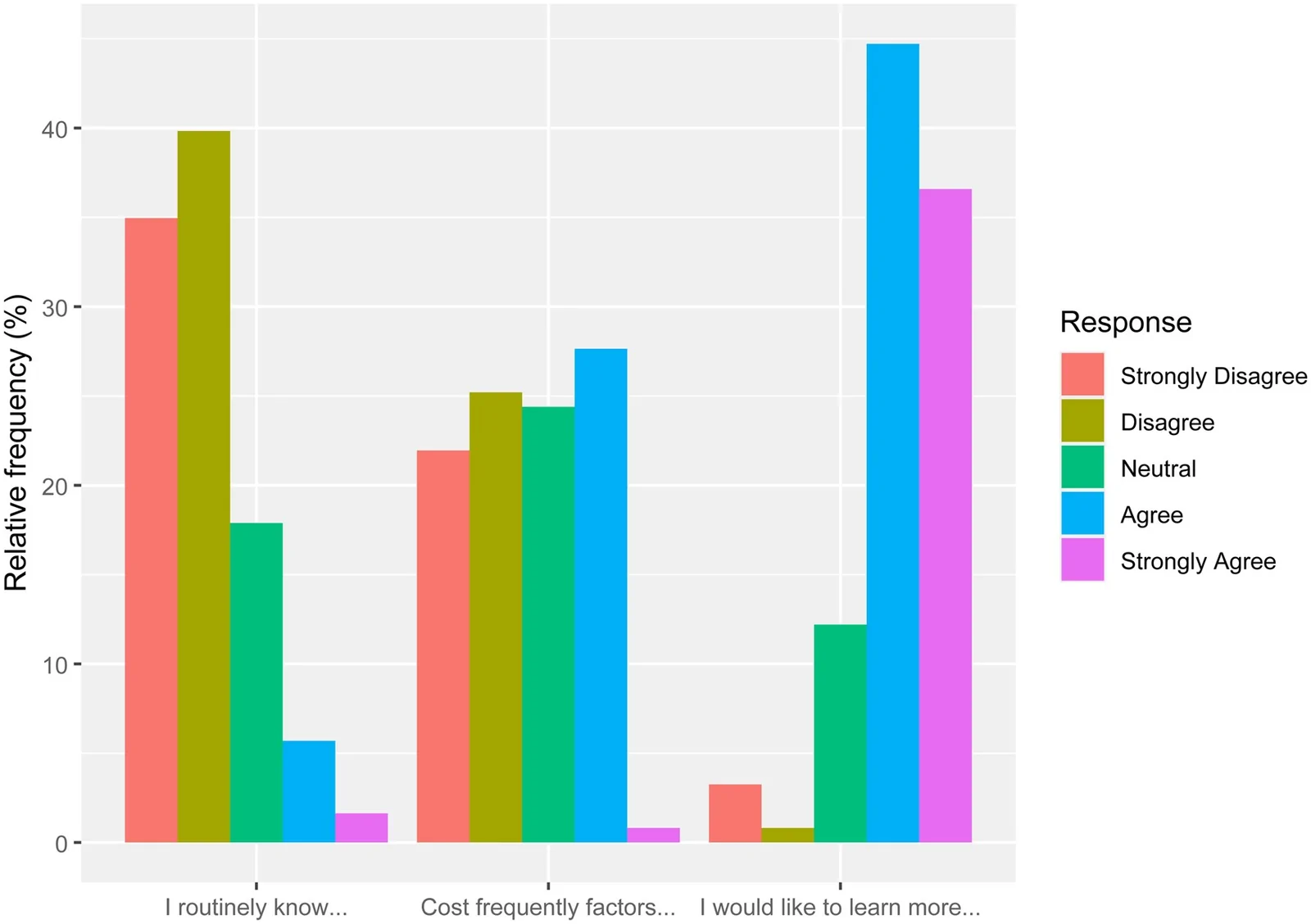
Likert scale responses to survey questions for self-assessment of surgical cost literacy.

Self-assessed knowledge of surgical supply costs was generally low. Although a majority of respondents disagreed with the statement that they routinely know the cost of supplies, most expressed interest in learning more (**Figure 1**).

**Figure 2.**
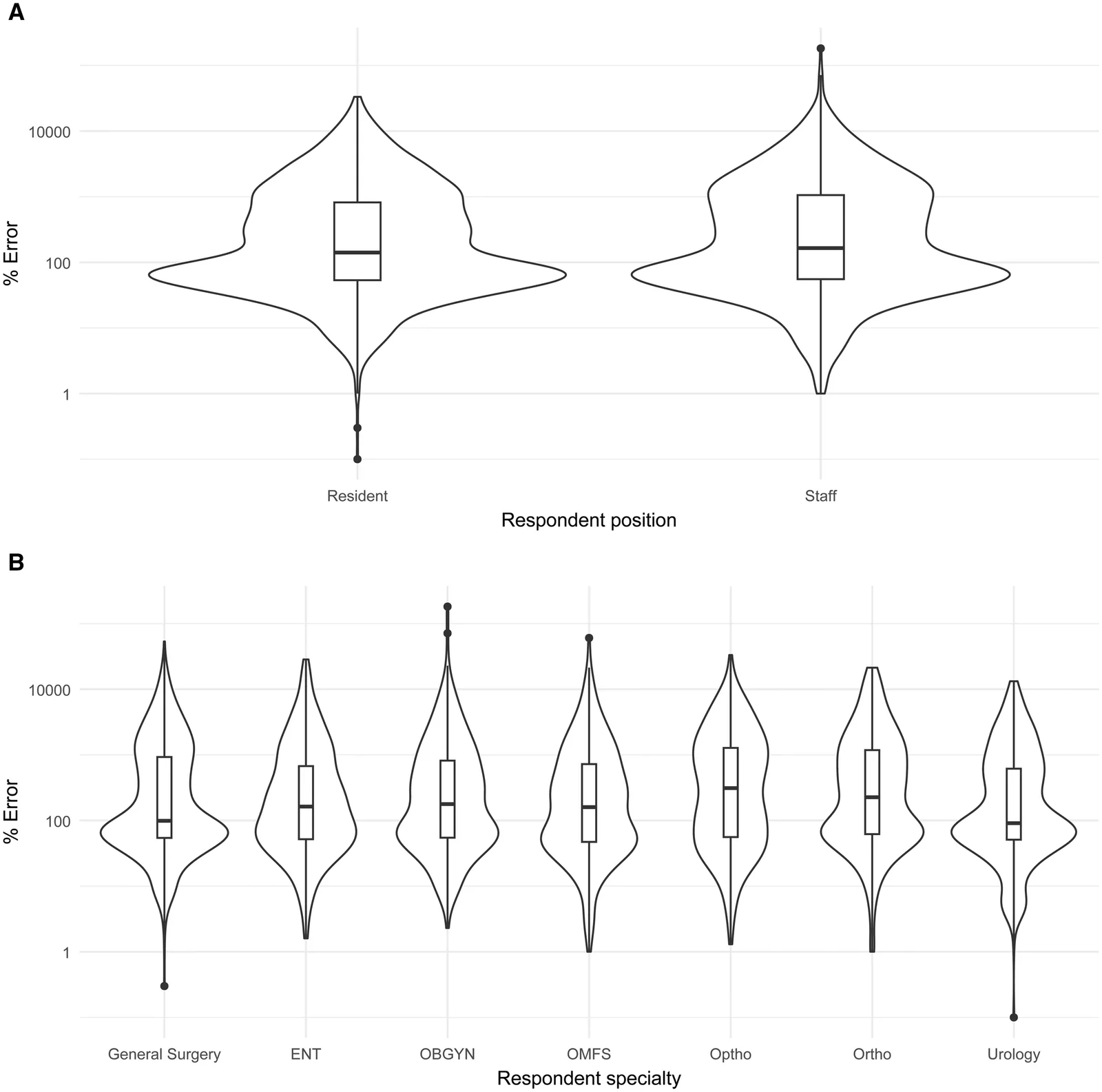
Violin plots of percent error by (A) position and (B) specialty.

Overall accuracy was 22.9%, with median error of 156.4% (IQR 53.9-891.7%). Median error for residents was 139.3% (IQR 53.3-817.4%), while median error for staff was 164.6% (IQR 55.4-1057.4%) (**Figure 2**). Estimate distributions for all common items were significantly different than true costs (**Table S1**). There were fewer significantly different estimate distributions for specialty-specific items (**Tables S2-S8**). On multivariable logistic regression, no significant differences in accuracy were found by age, sex, position, or experience length. Most specialties also did not differ significantly in accuracy except for orthopedic surgery, which was significantly less accurate (ref. general surgery; OR 0.59, 95% CI 0.4-0.86, *P *= .00674) (**Table 2**).

**Table 2.** Multivariable Logistic Regression for Accuracy

		OR [95% CI]	*P*-value
Age (per year of age)		0.99 [0.96-1.01]	0.351
Sex (ref. F)	M	0.99 [0.79-1.26]	0.965
Specialty (ref. general surgery)	ENT	1.04 [0.74-1.45]	0.832
	OBGYN	0.89 [0.62-1.27]	0.526
	OMFS	1.28 [0.88-1.84]	0.187
	Ophtho	0.99 [0.71-1.37]	0.934
	Ortho	0.59 [0.4-0.86]	0.00674
	Urology	1.02 [0.76-1.38]	0.876
Position (ref. Resident)	Staff	1.02 [0.75-1.37]	0.920
Experience length (per year of post-graduate experience)		1.01 [0.97-1.05]	0.605

ENT - ear, nose and throat; OBGYN - obstetrics and gynecology; OMFS - oral maxillofacial surgery; CI - confidence interval; M - male; F - female

## DISCUSSION

This study demonstrates a general lack of cost awareness among surgical staff and trainees, echoing prior literature.^3–7^ The breadth of our survey across multiple specialties and across the entire spectrum of surgical experience, however, provides additional insight into surgical cost awareness. Although one might expect that cumulative, incidental exposure to surgical costs would culminate in improved cost awareness and accuracy over the course of one’s career, we found that accuracy did not improve with theoretically more experienced staff surgeons compared to trainees and failed to improve with experience length in general. This suggests that informal exposure over time is insufficient for improving cost awareness and may reflect a need for more directed training such as the Choosing Wisely Canada program.^9^

We found that orthopedic surgery respondents were significantly less accurate than other specialties. It is difficult to speculate as to the reasons for this difference. The true costs of orthopedic implants and disposables spanned a vast range ($0.14-$10,124), which may have amplified estimation variability. This was not a feature specific to orthopedics, however, as specialties such as otolaryngology ($0.14-$14,200) and urology ($0.14-$12,563) also demonstrated significant ranges in cost. One possible explanation might be the prevalence of implantable hardware and kits in orthopedic surgery—we speculate that such implantable supplies may be associated with increased regulatory requirements and development/production costs and therefore may be more likely to be underestimated in cost than non-implantable supplies.

As our data were derived from a single-institution military setting, our findings may be limited in generalizability. There may also be components of response bias that are unaccounted for in our analysis and are inherent to our survey-based study design. There are, however, some advantages to our study setting—namely, the equal-access, single-payer military health system wherein physician and hospital reimbursement is completely uncoupled from services rendered. Institutional billing practices are therefore less likely to be a confounding factor in our study, as military personnel do not directly encounter cost data in the course of routine medical care. This institutional context undoubtedly influences our findings, and it is reasonable to hypothesize that a similar study performed in a private practice setting might yield greater cost awareness because of the more immediate impact of an item’s cost to such a practice’s bottom line. Indeed, one study of Saudi dermatology practices found that dermatologists who worked in mixed public-private practices were significantly more likely to be cost-consciousness positive compared to those who worked exclusively in government practice.^10^ The mixed public-private cohort, however, was still only found to have a 23% cost-consciousness positive rate, underscoring the broader fact that cost considerations in healthcare are generally poor irrespective of physician stratification.

Poor healthcare cost literacy has also been demonstrated in civilian, mixed-payer systems (namely Yale University, University of Pennsylvania, University of Maryland, and numerous others), suggesting that lack of billing exposure is far from the only driver of poor cost literacy in the healthcare system at large.^3–7^ The limitations in generalizability owing to the military setting are also offset by the breadth of specialties included in this study, making our findings relevant to all surgical professionals irrespective of degree of sub-specialization.

Areas for further investigation include expansion into non-surgical specialties as well as implementation of educational interventions with the aim of improving cost literacy through standardized, validated measures. Emerging literature suggests that such interventions may be effective; for instance, a software-based real-time feedback system was shown to significantly reduce the cost of disposable surgical supplies across multiple departments in one multispecialty trial.^11^ Similarly, another study which implemented real-time displays of intraoperative costs during thoracoscopic lobectomy yielded substantial per-case savings without compromising perioperative outcomes.^12^ We advocate for all surgeons, regardless of specialty or degree of experience, to seek out other potential avenues of improving cost literacy in order to continue delivering quality care while reducing the financial burden on patients and healthcare systems alike.

## CONCLUSION

This cross-sectional survey study across multiple surgical departments found low awareness of surgical supply costs among all specialties and degrees of career experience. These findings suggest a need for formal education in cost awareness, potentially as part of GME systems-based practice curricula.

## Supplementary Material

usaf595_Supplementary_Data

## Data Availability

Data are available for public review at https://dataverse.harvard.edu/dataset.xhtml?persistentId=doi:10.7910/DVN/GCNZVO.

## INSTITUTIONAL CLEARANCE

Institutional approval granted by Public Affairs Office.
