# Supplementary material for: The Price Is Right: Assessing Surgical Cost Awareness of Residents and Staff Across Surgical Specialties at a Single Training Hospital
Source: Mil Med. 2025 Dec 13;191(9-10):e1790–5. doi: 10.1093/milmed/usaf595 (PMC13542617; doi:10.1093/milmed/usaf595)
Supplement: Supplementary file 1 [file usaf595_supplementary_data.zip › milmed_supplement.docx]

**Supplementary Materials – Index**

**Supplementary Appendices**

Survey instrument – Part 1: Self-perceived cost knowledge	Page 2

Survey instrument – Part 2.1: True costs, common items	Page 2

Survey instrument – Part 2.2: True costs, specialty-specific items	Page 2

Otolaryngology (ENT)	Page 2

General Surgery	Page 3

Obstetrics/gynecology (OBGYN)	Page 4

Oral/Maxillofacial Surgery (OMFS)	Page 4

Ophthalmology	Page 5

Orthopedics	Page 6

Urology	Page 6

**Supplementary Figures and Tables**

Wilcoxon signed-rank testing, common items	Page 7

Wilcoxon signed-rank testing, specialty-specific items	Page 7

**Supplementary Appendices**

*Survey Instrument*

Part 1: Self-perceived cost knowledge

1.	I routinely know the surgical supplies cost of my cases.

2.	Cost frequently factors into my decision for operating room supplies.

3.	I would like to learn more about surgical supplies cost.

Responses ranged from 1 (strongly disagree) to 5 (strongly agree)

Part 2.1: True costs, common items

| Item | Cost of item (the dollar amount it costs to order the item for the department) |
| --- | --- |
| 1. Tegaderm Dressing 2 3/8 x 2 3/4 in | 0.33 |
| 2. Catheter Syringe Tip | 0.39 |
| 3. Size 8 surgical gloves | 1.66 |
| 4. Telfa Pad 3 x 4 in | 0.14 |
| 5. 22 g hypo needle | 0.15 |
| 6. 1,000 mL bag of IV Sodium Chloride | 1.52 |
| 7. IV start Kit | 1.21 |
| 8. Blood Pressure Cuff, adult, Large | 4.32 |
| 9. Surgical Gown XL | 11.34 |
| 10. Half Sheet (drape) | 1.09 |

Part 2.2: True costs, specialty-specific items

*Otolaryngology (ENT)*

| Item (one unit) | Cost of item (the dollar amount it costs to order the item for the department) |
| --- | --- |
| 1. Cochlear Implant Dual N7 Processor Single Unit (Device Only) – Cochlear | 14,200 |
| 2. Sinus Balloon SEEKR – Medtronic | 1,170 |
| 3. Microdebrider Quadcut Blade – Medtronic | 429 |
| 4. Propel Mini Stent Implant – Intersect ENT | 1,173.93 |
| 5. Coblator Turbinate Wand – Smith and Nephew | 239 |
| 6. HA Lite Partial Ossicular Replacement Prosthesis (PORP) – Grace Medical | 417 |
| 7. Stapes Piston Prosthesis – Grace Medical | 246 |
| 8. Shiley Tracheostomy Tube, size 6, with disposable inner cannula bevel tip – Covidien | 58.37 |
| 9. Ambu disposable flexible endoscope | 190 |
| 10. Doyle Septal Splints – Medtronic | 21.9 |

*General Surgery*

| Item (per unit) | Cost of item (the dollar amount it costs to order the item for the department) |
| --- | --- |
| 1. Laparoscopic Stapler Reloads – (Tan 45 mm) - Endo GIA | 182.24 |
| 2. 5mm Laparoscopic Ligasure Device, Maryland - Covidien | 599.86 |
| 3. Ligasure Impact Device curved/large jaw – Covidien | 692.17 |
| 4. Contour Curved Stapler Blue – Ethicon | 464.72 |
| 5. Progrip mesh for IHR Laparoscopic – Medtronic | 340.45 |
| 6. Robotic Green reloads (45 mm) – Intuitive Surgical | 187.48 |
| 7. Carter-Thomason closure device – Cooper Surgical | 135.45 |
| 8. Robotic arm drape – Intuitive Surgical | 54.7 |
| 9. Robotic Suction irrigator (8mm) – Intuitive Surgical | 272.92 |
| 10. Parietex composite mesh 10x15cm – Medtronic | 430.53 |

*Obstetrics/gynecology (OB/GYN)*

| Item (per unit) | Cost of item (the dollar amount it costs to order the item for the department) |
| --- | --- |
| 1. Myosure Lite Disposable Hysteroscopic Polyp Removal Device - Hologic | 671.75 |
| 2. Word Catheter (for Bartholin’s) | 20.5 |
| 3. Arista 1gm Single Use – Bard | 107.44 |
| 4. Alexis retractor (XL) – Applied Medical Resources | 90.78 |
| 5. PhotonSaber Lighted Suction Device – Stryker | 503.82 |
| 6. Endoloop – Ethicon | 46.14 |
| 7. Ligasure Impact – Covidien | 692.17 |
| 8. Postpartum Abdominal Binder XL – DeRoyal | 14.81 |
| 9. Pink Pad (for t-burg) – Xodus Medical | 110.61 |
| 10. Disposable Clip Applier, Large, 20 Clips – Conmed | 116.69 |

*Oral Maxillofacial Surgery (OMFS)*

| Item (each) | Cost of item (the dollar amount it costs to order the item for the department) |
| --- | --- |
| 1. Lidocaine Carpules 1.7 ml, 2% w/ epi – Henry Schein | 0.57 |
| 2. 3.0 chromic gut suture – Ethicon | 2.93 |
| 3. 1.2cc particulate bone graft – Stryker | 85.66 |
| 4. Osseotite Endosseous Implant 4x11.5 – Biomet | 304.89 |
| 5. Microplate Maxillary Fixation L-shape plate – KLS Martin | 223.22 |
| 6. Locking Mandibular Angle Plate – KLS Martin | 1040.26 |
| 7. O2 Nasal cannula Tubing | 4.49 |
| 8. Cytoplast Type I Bovine Collagen membrane – Osteogenics Biomedical | 152.53 |
| 9. Maxdrive MiniScrew Fixation Screw 2x7mm – KLS Martin | 64.36 |
| 10. 4.3mm Carbide Burrs | 4.86 |

*Ophthalmology*

| Item (per unit) | Cost of item (the dollar amount it costs to order the item for the department) |
| --- | --- |
| 1. Technic ZCB00 Intraocular lens Implant – Johnson & Johnson | 93.75 |
| 2. 1020 Drape – Alcon | 6.1 |
| 3. Centurion Phacoemulsification Pack – Alcon | 153.19 |
| 4. Disposable 4-Mirror Gonioscopy Lens – Volk | 12.11 |
| 5. Disposable Jeweler’s Forceps (Fine Angled) – BVI Medical | 16.5 |
| 6. Vigamox (Moxifloxacin) Antibiotic Drops (3 ml Dropper) – Novartis | 7.58 |
| 7. Symfony ZXR00 ERV Intraocular Lens Implant – Johnson & Johnson | 850 |
| 8. Iris Hooks – Alcon | 72.25 |
| 9. 7.0 mm Malyugin Ring Iris Expansion Device – Microsurgical Technology INC | 133.17 |
| 10. Miochol for Intraocular Use (20mg/2ml Kit) – Bausch+Lomb | 57.36 |

*Orthopedics*

| Item | Cost of item (the dollar amount it costs to order the item for the department) |
| --- | --- |
| 1. Total Knee Arthroplasty – Zimmer | 5050.61 |
| 2. Total Hip Arthroplasty – Stryker | 1200.68 |
| 3. Hemi-arthroplasty Hip – Intellijoint | 1445 |
| 4. Digit Widget – Hand Bio Mechanics Lab | 1650 |
| 5. Osteochondral Allograft – Stryker | 3319.28 |
| 6. Cervical Disk Arthroplasty, Cervical Mobi-C Radiculopathy – Zimmer | 10123.92 |
| 7. Variable Pitched Scaphoid Screw – Trimed | 619.2 |
| 8. Knotless Fibertac – Arthrex | 432 |
| 9. Semi-tendinosis Allograft – Cardinal Health | 3255.7 |
| 10. Metatarsal Bone Graft Kit - Stryker | 3319.28 |

*Urology*

| Item | Cost of item (the dollar amount it costs to order the item for the department) |
| --- | --- |
| 1. Testicular Implant (coloplast) | 1,953.94 |
| 2. Pigtail Stent (cook Medical) | 157.44 |
| 3. Sensor Wire (Boston Scientific) straight tip | 47.57 |
| 4. Single Action Pump (boston scientific) | 52.63 |
| 5. Altis Single Incision Sling (coloplast) | 1,563.15 |
| 6. AMS, AUS (boston Scientific) | 7,614.52 |
| 7. 3.9mm X 440mm Trilogy Probe Kit (Boston Scientific) | 1,314.80 |
| 8. Boston Scientific 11/13 access sheath | 211.15 |
| 9. AMS Boston Scientific IPP | 12,563.41 |
| 10. Dakota Retrieval Basket (boston Scientific) | 368.14 |

**Supplementary Figures and Tables**

*Table S1. Wilcoxon signed-rank testing, common items*

|  | variable | statistic | p.value | method | alternative |
| --- | --- | --- | --- | --- | --- |
| 1 | Item 1 | 7501 | 8.16E-22 | Wilcoxon signed rank test | two.sided |
| 2 | Item 2 | 7349 | 2.60E-21 | Wilcoxon signed rank test | two.sided |
| 3 | Item 3 | 7422 | 6.33E-21 | Wilcoxon signed rank test | two.sided |
| 4 | Item 4 | 7503 | 8.25E-22 | Wilcoxon signed rank test | two.sided |
| 5 | Item 5 | 7497 | 9.66E-22 | Wilcoxon signed rank test | two.sided |
| 6 | Item 6 | 7601 | 1.07E-21 | Wilcoxon signed rank test | two.sided |
| 7 | Item 7 | 7620 | 6.85E-22 | Wilcoxon signed rank test | two.sided |
| 8 | Item 8 | 7521 | 7.46E-21 | Wilcoxon signed rank test | two.sided |
| 9 | Item 9 | 6282 | 4.49E-10 | Wilcoxon signed rank test | two.sided |
| 10 | Item 10 | 7497 | 9.73E-22 | Wilcoxon signed rank test | two.sided |

*Wilcoxon signed-rank testing, specialty-specific items*

*Table S2. Otolaryngology (ENT)*

|  | variable | statistic | p.value | method | alternative |
| --- | --- | --- | --- | --- | --- |
| 1 | Item 11 - ENT | 88 | 0.025442 | Wilcoxon signed rank test | two.sided |
| 2 | Item 12 - ENT | 43 | 0.549939 | Wilcoxon signed rank test | two.sided |
| 3 | Item 13 - ENT | 56 | 0.826007 | Wilcoxon signed rank test | two.sided |
| 4 | Item 14 - ENT | 1 | 0.001205 | Wilcoxon signed rank test | two.sided |
| 5 | Item 15 - ENT | 67 | 0.362094 | Wilcoxon signed rank test | two.sided |
| 6 | Item 16 - ENT | 50 | 0.87523 | Wilcoxon signed rank test | two.sided |
| 7 | Item 17 - ENT | 90 | 0.018451 | Wilcoxon signed rank test | two.sided |
| 8 | Item 18 - ENT | 99 | 0.003462 | Wilcoxon signed rank test | two.sided |
| 9 | Item 19 - ENT | 73 | 0.19757 | Wilcoxon signed rank test | two.sided |
| 10 | Item 20 - ENT | 99 | 0.003343 | Wilcoxon signed rank test | two.sided |

*Table S3. General Surgery*

|  | variable | statistic | p.value | method | alternative |
| --- | --- | --- | --- | --- | --- |
| 1 | Item 11 - Gen Surg | 202 | 0.367032 | Wilcoxon signed rank test | two.sided |
| 2 | Item 12 - Gen Surg | 70 | 0.000479 | Wilcoxon signed rank test | two.sided |
| 3 | Item 13 - Gen Surg | 51 | 0.000112 | Wilcoxon signed rank test | two.sided |
| 4 | Item 14 - Gen Surg | 100 | 0.003712 | Wilcoxon signed rank test | two.sided |
| 5 | Item 15 - Gen Surg | 253 | 0.921873 | Wilcoxon signed rank test | two.sided |
| 6 | Item 16 - Gen Surg | 355 | 0.035871 | Wilcoxon signed rank test | two.sided |
| 7 | Item 17 - Gen Surg | 97 | 0.003064 | Wilcoxon signed rank test | two.sided |
| 8 | Item 18 - Gen Surg | 340 | 0.070897 | Wilcoxon signed rank test | two.sided |
| 9 | Item 19 - Gen Surg | 102 | 0.00417 | Wilcoxon signed rank test | two.sided |
| 10 | Item 20 - Gen Surg | 259 | 0.829228 | Wilcoxon signed rank test | two.sided |

*Table S4. Obstetrics/gynecology*

|  | variable | statistic | p.value | method | alternative |
| --- | --- | --- | --- | --- | --- |
| 1 | Item 11 - OBGYN | 50 | 0.875169 | Wilcoxon signed rank test | two.sided |
| 2 | Item 12 - OBGYN | 71 | 0.245028 | Wilcoxon signed rank test | two.sided |
| 3 | Item 13 - OBGYN | 53 | 0.974867 | Wilcoxon signed rank test | two.sided |
| 4 | Item 14 - OBGYN | 81 | 0.072885 | Wilcoxon signed rank test | two.sided |
| 5 | Item 15 - OBGYN | 20 | 0.074203 | Wilcoxon signed rank test | two.sided |
| 6 | Item 16 - OBGYN | 47 | 0.729571 | Wilcoxon signed rank test | two.sided |
| 7 | Item 17 - OBGYN | 19 | 0.035148 | Wilcoxon signed rank test | two.sided |
| 8 | Item 18 - OBGYN | 99 | 0.003462 | Wilcoxon signed rank test | two.sided |
| 9 | Item 19 - OBGYN | 71 | 0.244795 | Wilcoxon signed rank test | two.sided |
| 10 | Item 20 - OBGYN | 84 | 0.046619 | Wilcoxon signed rank test | two.sided |

*Table S5. Oral/maxillofacial surgery*

|  | variable | statistic | p.value | method | alternative |
| --- | --- | --- | --- | --- | --- |
| 1 | Item 11 - OMFS | 74 | 0.005847 | Wilcoxon signed rank test | two.sided |
| 2 | Item 12 - OMFS | 70 | 0.014945 | Wilcoxon signed rank test | two.sided |
| 3 | Item 13 - OMFS | 63 | 0.05915 | Wilcoxon signed rank test | two.sided |
| 4 | Item 14 - OMFS | 32 | 0.582047 | Wilcoxon signed rank test | two.sided |
| 5 | Item 15 - OMFS | 61 | 0.083782 | Wilcoxon signed rank test | two.sided |
| 6 | Item 16 - OMFS | 24 | 0.238593 | Wilcoxon signed rank test | two.sided |
| 7 | Item 17 - OMFS | 59 | 0.115961 | Wilcoxon signed rank test | two.sided |
| 8 | Item 18 - OMFS | 23 | 0.208549 | Wilcoxon signed rank test | two.sided |
| 9 | Item 19 - OMFS | 41 | 0.875186 | Wilcoxon signed rank test | two.sided |
| 10 | Item 20 - OMFS | 52 | 0.307076 | Wilcoxon signed rank test | two.sided |

*Table S6. Ophthalmology*

|  | variable | statistic | p.value | method | alternative |
| --- | --- | --- | --- | --- | --- |
| 1 | Item 11 - Ophtho | 120 | 0.000648 | Wilcoxon signed rank test | two.sided |
| 2 | Item 12 - Ophtho | 104 | 0.001208 | Wilcoxon signed rank test | two.sided |
| 3 | Item 13 - Ophtho | 82 | 0.063843 | Wilcoxon signed rank test | two.sided |
| 4 | Item 14 - Ophtho | 102 | 0.001858 | Wilcoxon signed rank test | two.sided |
| 5 | Item 15 - Ophtho | 73.5 | 0.186749 | Wilcoxon signed rank test | two.sided |
| 6 | Item 16 - Ophtho | 105 | 0.000976 | Wilcoxon signed rank test | two.sided |
| 7 | Item 17 - Ophtho | 64 | 0.469681 | Wilcoxon signed rank test | two.sided |
| 8 | Item 18 - Ophtho | 57 | 0.777029 | Wilcoxon signed rank test | two.sided |
| 9 | Item 19 - Ophtho | 33 | 0.219531 | Wilcoxon signed rank test | two.sided |
| 10 | Item 20 - Ophtho | 39 | 0.394974 | Wilcoxon signed rank test | two.sided |

*Table S7. Orthopedics*

|  | variable | statistic | p.value | method | alternative |
| --- | --- | --- | --- | --- | --- |
| 1 | Item 11 - Ortho | 107 | 0.14845 | Wilcoxon signed rank test | two.sided |
| 2 | Item 12 - Ortho | 148 | 0.000705 | Wilcoxon signed rank test | two.sided |
| 3 | Item 13 - Ortho | 144 | 0.001382 | Wilcoxon signed rank test | two.sided |
| 4 | Item 14 - Ortho | 17.5 | 0.005135 | Wilcoxon signed rank test | two.sided |
| 5 | Item 15 - Ortho | 152 | 0.000342 | Wilcoxon signed rank test | two.sided |
| 6 | Item 16 - Ortho | 59 | 0.406777 | Wilcoxon signed rank test | two.sided |
| 7 | Item 17 - Ortho | 63 | 0.521436 | Wilcoxon signed rank test | two.sided |
| 8 | Item 18 - Ortho | 72 | 0.83117 | Wilcoxon signed rank test | two.sided |
| 9 | Item 19 - Ortho | 19 | 0.00646 | Wilcoxon signed rank test | two.sided |
| 10 | Item 20 - Ortho | 5 | 0.000698 | Wilcoxon signed rank test | two.sided |

*Table S8. Urology*

|  | variable | statistic | p.value | method | alternative |
| --- | --- | --- | --- | --- | --- |
| 1 | Item 11 - Uro | 29 | 0.004504 | Wilcoxon signed rank test | two.sided |
| 2 | Item 12 - Uro | 78 | 0.313126 | Wilcoxon signed rank test | two.sided |
| 3 | Item 13 - Uro | 187 | 0.002153 | Wilcoxon signed rank test | two.sided |
| 4 | Item 14 - Uro | 149 | 0.098768 | Wilcoxon signed rank test | two.sided |
| 5 | Item 15 - Uro | 61 | 0.100251 | Wilcoxon signed rank test | two.sided |
| 6 | Item 16 - Uro | 40 | 0.015196 | Wilcoxon signed rank test | two.sided |
| 7 | Item 17 - Uro | 52 | 0.047431 | Wilcoxon signed rank test | two.sided |
| 8 | Item 18 - Uro | 47 | 0.030293 | Wilcoxon signed rank test | two.sided |
| 9 | Item 19 - Uro | 15 | 0.000772 | Wilcoxon signed rank test | two.sided |
| 10 | Item 20 - Uro | 42 | 0.018593 | Wilcoxon signed rank test | two.sided |
